# False Impressions

**Published:** 1889-02-23

**Authors:** Caroline Fothergill

**Affiliations:** Author of "An Enthusiast," "A Voice in the Wilderness," etc.


### FALSE IMPRESSIONS.

By Caroline Fothergill, Author of "An Enthusiast," "A Voice in the Wilderness," etc.

*(Continued from p. 322.)*

### Chapter XX.—Mischief.

By this time almost everyone in Mayfield had gone away from home. Edith wondered if she would go away. Mayfield was very hot and dusty, but her aunt said nothing about any change; so Edith did not expect any, although she was beginning to droop a little.

It was a fortnight since Mrs. Lester had taken her resolution, and she had not yet seen an opportunity for giving Edith the hints she intended. She was growing a little anxious, though she had detected no sign that George's marriage was any nearer at hand. But in the long run an opportunity for doing evil is never wanting, and the occasion she desired presented itself before long.

For a week or ten days the heat had been intense. Edith, naturally delicate and without much power of endurance, bore up for a day or two, and then broke down and was reduced to going to bed, and spending her days in lounging upon couches, as Mrs. Lester called it. She was sorry for her niece, and was kind to her in a sour, severe way, which Edith bore in meek silence, and wept over when she was alone and unobserved. George was very sorry to see her utter prostration; he was genuinely kind; brought her fruit and books, carried her up and down stairs, sat for an hour at a time chatting with her, and was altogether as kind as it was possible to be. Neither noticed how much they were left together, or how frequently Mrs. Lester suggested that George should amuse Edith, or peel grapes and peaches for her. George would have done half these things of his own accord, because he was fond of Edith and glad to give her pleasure, and Edith lay in a kind of half-dream of perfect happiness, conscious only that George was constantly with her, that his deep, grave voice answered her almost whenever she spoke, that his cool, firm hand often held her little fevered fingers. She thought that was happiness enough, and bore her weakness almost cheerfully because of these indulgences; but there was greater happiness to come.

One evening, the close of a hot, airless, and thundery day, Edith felt particularly depressed. She lay on her couch with closed eyes, and George, who had been reading to her, was now silent under the impression that she slept. Mrs. Lester was not in the room; the whole house was perfectly still—oppressively still; any sound would have been welcome. Edith was not asleep, only thinking, and somehow her thoughts had gone back to her dingy London home, to her hard-worked, careworn father, and many rough and romping brothers. Although, had she been amongst them in her present state, she would have been utterly overwhelmed and helpless, the thought of them, softened by time and distance and in her present weak state, was too much for her; she felt as if she would give all the luxuries of Queen Street for half-an-hour in the old patched and shabby dining-room with its darned carpet, and the huge unwieldy armchair belonging to her father, and irreverently nicknamed "The Bus" by the younger boys. In Queen Street the furniture was in perfect repair, the couch upon which she lay was deliciously comfortable and restgiving, while "The Bus" was literally out at elbows, and the leather covering hung in what the old servant called "chags" from the seat; but just now Edith felt, as if to curl herself up in the depths of that chair and lean her head against the back, were it but for half-an-hour, would make her perfectly strong again. Her eyes filled, her mouth trembled, the tears began to roll down her cheeks. When she tried to restrain them she found that all her self-control was gone; they only came faster with every effort she made to stop them. She was forced to get her handkerchief to wipe them away, and the movement drew George's attention to the fact that she was not asleep. He spoke to her, but received no answer; and the reply to his second question was a deep sob. The floodgates were opened now, and there was nothing for it but to let her have her cry out. George behaved very kindly; he sent away the maid, who just then came with lights, and sat silent by her until she was calm enough to speak. When she had reached that stage he rang for the lamps, and began to speak cheerfully of a plan which he said had just occurred to him, and which he felt sure would put Edith into perfectly good spirits again. It was clear, he said, that she was thoroughly run down, and needed more change than could be got from lying on the sofa, one half of the day in the morning-room and the other in the drawing-room. He proposed, therefore, that the whole family of them—Mrs. Lester, Edith, and himself—should go to Switzerland as soon as ever arrangements could be made: the next day if possible.

Edith heard in raptured silence, her little thin hands tightly clasped upon her breast, her large eyes shining through her unshed tears. She dared not think such happiness as this possible, and she listened for her aunt's voice, in miserable certainty that as soon as her brother had finished speaking it would be uplifted in condemnation of the scheme, on the ground of either the expense, or the distance, or the trouble, or the general undesirability of the whole thing. But, no! Edith could scarcely believe she heard aright when Mrs. Lester began to speak suavely of the immense pleasure it would give her to revisit Switzerland, and the good she thought it would do Edith, both in bracing her up, and also in bringing her out more and giving her greater confidence of manner. Edith's breath almost failed her at these announcements. When before had her aunt expressed any wish that she might be "brought out more," and what was the meaning of the change?

Mrs. Lester also revealed, with a readiness of invention which did her infinite credit, that she had thought of taking Edith herself, but that ladies travelling alone got so little attention nowadays, and she had not liked to trespass on her brother's time and freedom so far as to ask him to accompany them. Now that he himself suggested it, she seconded the proposal heartily. They had no need to ask Edith's opinion, her face was enough; and George laughed at the stammering words in which she tried to express her pleasure and gratitude. He began to draw up a kind of plan of their journey, mentioning things, and scenes, and places which he would like Edith to see; and as he could be away only a month, he wanted to get in as much as possible without overtaxing Edith's strength. He thought they would not do more than the Bernese Oberland. That was enough for one journey, and it was not the only journey he hoped they would take together. They could leave the Engadine for another summer, when he might arrange to be away a longer time. Then he patted Edith's hand, and told her she had had enough excitement for one evening; she had better go to bed, and leave her aunt and himself to talk over the details and see how soon they could get away.

Edith would have been ready to start off then and there, with or without her wardrobe, and she was almost disappointed the next morning to hear that they were not going that very day, but must wait until the next, that George might make some necessary arrangements.

In the afternoon Harry Fairfax called. He had heard of Edith's breakdown, and came full of sympathy and kindness He brought an offering with him in the shape of a basket of fruit; but Edith, rather spoilt by George's indulgence, scarcely looked at it, although she thanked him very prettily for his kind thought in bringing it. He told her about his visits to Mrs. Jones, and how it was from her he had first heard of Edith's illness, and he related various bits of parish news which he thought would interest her.

She listened, but absently, as though she were not paying much attention to what he said; and as soon as an opening came she began to pour out her tale about the Swiss journey. She spoke with such enthusiasm and almost emotion of Mr. Murray's kindness that Harry was a little puzzled. But for the great discrepancy between their ages, positions, and characters, he might have made a sudden guess at the truth. But he put such a thought away from him as being disrespectful to Edith, and listened to her rapturous outburst with as much sympathy as he could summon up; but he could not altogether repress a feeling of bitterness that Mr. Murray, who was nothing to Edith, should be able to do so much for her, while he, who would give his life for her, could do practically nothing.

In the evening Mrs. Lester and Edith were alone. The packing was nearly finished; they would start at ten o'clock the next morning. Mrs. Lester, who retained her amiability of the previous evening, had been telling Edith the route plan for the first day or two, and Edith was again speaking of Mr. Murray's kindness. When she had subsided into silence, Mrs. Lester saw that her opportunity had come, and took advantage of it.

"Edith, my dear," she began, as she cleared her throat, "before we start, I should like to give you a little word of warning."

"Yes, aunt," said Edith, a little startled; "what about?"

"Try to lay a little restraint upon the way in which you speak to and act with my brother. Amongst ourselves it is all very well; we understand one another; but strangers might think it odd. When we live in hotels we should always be a little guarded in our conduct."

"I don't understand, aunt; what do you mean? Have I done anything wrong?" asked Edith, stammering and changing colour in her confusion and consciousness.

"No, my dear, nothing absolutely wrong," answered Mrs. Lester, pleasantly. "Only you show your feelings a little too plainly, and in a girl of your age that is not wise."

"Aunt, what do you mean?" repeated Edith in some agitation.

"Well, it is not difficult to explain my meaning. Anyone with a little more experience would have understood me at once. To anyone else I should have spoken sooner, but I did not want to open your eyes too soon. Innocence in these things is a great attraction to many men, especially men who have seen a good deal of life, as my brother has."

Edith sat up, white and trembling.

"Oh, aunt," she said, imploringly, "please speak out. I don't know what you mean, indeed. What has Mr. Murray to do with it?"

Mrs. Lester laid down her book, and went straight into what she wanted to say.

"You often speak of my brother's kindness to you—and he has been very kind. If you knew how little notice he takes of young girls as a rule, you would better understand what an exception he has made in your favour. Has it never come into your mind to ask yourself why he is so kind?"

There was such a significance in her tone and look that Edith went first crimson and then white, and felt as if she did not know where she was.

"You understand now what I mean," she said with a smile.

It was impossible not to understand, and Edith trembled all over, and felt as if she would faint with mingled joy and fear. She had long known that she loved George, but she had never entertained the hope that George would love her.

"So you will agree with me," went on her aunt suavely, "when I say you had better lay a little restraint upon your manner with my brother. When we are among strangers they will soon see what I have pointed out to you, and it will only cause amusement if you show your feelings so plainly. George, too, will like you all the better for a little reticence.'

"But, aunt, are you sure?" asked Edith, as soon as she could command her voice sufficiently to speak.

Mrs. Lester smiled mysteriously.

"George has said nothing of course—he is very reticent; but I have eyes, and can see as clearly as my neighbours."

"And—and you would not dislike it?" asked Edith timidly, and not meeting her aunt's eyes.

"Well, if I had disliked it, I should scarcely have talked to you this evening as I have done. We have never quarrelled yet, and you would not refuse me my corner by the fire, would you?"

"Oh, aunt, how can you speak in that certain tone?" said Edith, almost dismayed, and too thoroughly overwhelmed and confused to notice the unnatural way in which Mrs. Lester was talking. "I cannot believe it," she said after a pause: "it seems so strange. I am such a poor little thing."

"Not so very strange," said her aunt briskly. "You are a very pretty girl, and you have always suited George, or he would not have taken you out as much as he has done."

Mrs. Lester had never spoken of her brother as "George" to Edith before, and the girl was much impressed by the change. She listened to all that her aunt had still to say, and at last went to bed in a half-dream and wondering how she would meet George's eyes when next she saw him.

Mrs. Lester sat on, smiling to herself and saying—

"That was a clever move. If things are just where they are now when we come home again, it will be Edith's fault, not mine. If it is necessary I will tell him she is in love with him, and then he will feel bound to marry her. I know George."

*(To be continued.)*

